# A comprehensive quality assurance procedure for 4D CT commissioning and periodic QA

**DOI:** 10.1002/acm2.13764

**Published:** 2022-09-04

**Authors:** Mitchell Polizzi, Siyong Kim, Mihaela Rosu‐Bubulac

**Affiliations:** ^1^ Department of Radiation Oncology Virginia Commonwealth University Richmond Virginia USA

**Keywords:** 4D CT, CT simulation, quality assurance, radiotherapy, respiratory motion‐management

## Abstract

**Purpose:**

The 4D computed tomography (CT) simulation is an essential procedure for tumors exhibiting breathing‐induced motion. However, to date there are no established guidelines to assess the characteristics of existing systems and to describe meaningful performance. We propose a commissioning quality assurance (QA) protocol consisting of measurements and acquisitions that assess the mechanical and computational operation for 4D CT with both phase and amplitude‐based reconstructions, for regular and irregular respiratory patterns.

**Methods:**

The 4D CT scans of a QUASAR motion phantom were acquired for both regular and irregular breathing patterns. The hardware consisted of the Canon Aquilion Exceed LB CT scanner used in conjunction with the Anzai laser motion monitoring system. The nominal machine performance and reconstruction were demonstrated with measurements using regular breathing patterns. For irregular breathing patterns the performance was quantified through the analysis of the target motion in the superior and inferior directions, and the volume of the internal target volume (ITV). Acquisitions were performed using multiple pitches and the reconstructions were performed using both phase and amplitude‐based binning.

**Results:**

The target was accurately captured during regular breathing. For the irregular breathing, the measured ITV exceeded the nominal ITV parameters in all scenarios, but all deviations were less than the reconstructed slice thickness. The mismatch between the nominal pitch and the actual breathing rate did not affect markedly the size of the ITV. Phase and normalized amplitude binning performed similarly.

**Conclusions:**

We demonstrated a framework for measuring and quantifying the initial performance of 4D CT simulation scans that can also be applied during periodic QAs. The regular breathing provided confidence that the hardware and the software between the systems performs adequately. The irregular breathing data suggest that the system may be expected to capture in excess the target motion and geometry, but the deviation is expected to be within the slice thickness.

## 1 INTRODUCTION

The 4D computed tomography (CT) has been widely implemented in radiation oncology as a technique to create multiple anatomical 3D image datasets that characterize the patient's anatomy at various stages over the respiratory cycle. The accurate modeling of the anatomy is challenging, due to technical limitations of the scanner, irregularities in the patient breathing, uncertainties in the correlation between the breathing motion surrogate and the target motion, and the interplay between the CT scanner table and gantry movements on one side, and the tumor (or other organs of interest) movements on the other side. The accuracy of the extent of tumor motion is essential to ensure target coverage throughout the entirety of the breathing cycle. Discrepancies in the dosimetric coverage have been found when the tumor motion is large and irregular and cannot be accounted for with 3D CT and uniform margins.^1^ Understanding the boundaries of the expected accuracy is undeniably important, as it is their subsequent management, because the image artifacts in 4D may negatively influence processes in radiation oncology,^2^ and ultimately, the treatment outcome.^3^

The generation of breathing‐stage‐specific 3D CT image sets can be accomplished using prospective and retrospective strategies.

The prospective strategies all result in one single image set, corresponding to one breathing stage, being created. This is accomplished either by scanning the patient while motionless at that breathing stage (which necessitates voluntary or aided breath‐hold), or by allowing the patient to breath normally and acquiring the imaging data only when the patient is at the desired stage of the respiratory cycle (gated acquisition).

For the retrospective strategies, also referred to as 4D imaging, the patient breathes freely and the outcome of such a scanning procedure consists of a series of 3D image sets that describe the patient's anatomy at various breathing stages. The scan can be performed in either ciné^4^ or helical^5^ modes. In ciné mode, the couch is stationary while a spiral CT is acquired for a time equal to the duration of the breathing cycle plus the duration of a full gantry rotation, to ensure that data is acquired over at least one breathing cycle; then the couch moves to the next position and the process repeats. In helical mode, the couch moves continuously during the image acquisition, at a constant speed, set by a pitch factor (defined as the ratio between the distance the table translates in one gantry rotation and the width of the x‐ray collimation). Each section of the anatomy must be imaged over a full breathing cycle at each slice location, which is accomplished if the ratio between the gantry rotation time and pitch exceeds the breathing period.^6^

In retrospective scanning, each imaging projection is time‐tagged and associated with a breathing stage within the breathing cycle during which it was acquired. The 3D image sets corresponding to various breathing stages are then reconstructed by binning together only the projection data that corresponds to that breathing stage, with the binning being done by either phase or amplitude.^7^, ^8^, ^9^, ^10^, ^11^ These image sets can be used to identify the target at each breathing stage for real‐time tumor tracking treatment deliveries, or for ITV‐based treatment planning and delivery (ITV, the internal target volume, is defined as the union of the targets over all image sets available).

The difficulty in generating artifact‐free 4D image sets is rooted mainly in the temporo‐spatial variability of the breathing pattern. With the current CT technology available in the scanners typically used in radiation oncology, this respiratory variability cannot be dynamically matched, during the scanning procedure, by the gantry rotation and the couch travel speed. Moreover, the difficulties associated with predicting accurately the breathing pattern and with the system time of reaction lag—which are prerequisites for adjusting the table speed and gantry rotation time, would make the task very difficult even if the variable table speed was technically feasible.

Each binning method has been widely explored within the literature^8^, ^12^ and each method has their benefits and drawbacks. Amplitude binning has a theoretical benefit in that it reconstructs based upon the same “geometrical” level of the surrogate trace, reducing the artifacts due to “misplaced” anatomy. It has been found that image artifacts are reduced with amplitude binning, with one of the most common examples being the “zipper” artifact at the dome of the liver. However, amplitude binning may suffer from insufficient binning data points if the surrogate amplitude is irregular throughout the acquisition. For amplitude binning, if a reconstruction bin has data collected during every breathing cycle, the target volume from this image set should reproduce accurately the actual object volume. As noted earlier, amplitude binning is vulnerable to missing data, if there are bins that do not contain information in all amplitudes of the target motion. This may be alleviated through normalization, which will fill all the normalized amplitude bins with data, with the downside that it will lead to miss‐binning of the amplitude at various points in the acquisition where the amplitude was rescaled from its real value. Phase binning offers the benefit of access to all the data points, though the phases of breathing may not correspond to the target and anatomy in the same location due to irregular breathing patterns.

However, all these difficulties and the almost guaranteed inaccuracies in each individual image set generated through 4D acquisitions, do not negate the usefulness of 4D image sets in providing a patient‐specific representation of the breathing motion effect on the anatomy, which, even if not fully accurate, is still an important improvement toward customizing the radiation treatment margins to account for respiratory‐induced movements on a case‐by‐case basis.

All things considered, the 4D technology is here to stay and even though a patient‐specific image quality assurance (QA) is a difficult, if not impossible, endeavor, the 4D system QA should be an integral part of the CT QA at large.

The 4D system relies upon a respiratory signal provided by breathing motion surrogates that is correlated with the acquired projection data. The accuracy of the transfer of the breathing pattern to the computer that utilizes it for image reconstruction is essential to the quality of the process. Additionally, depending on the endpoint, the amplitude of motion for the target, the target volume and shape at each breathing phase, as well as the motion‐encompassing volume or ITV are essential. In this work, we propose a straightforward methodology to perform QA at the time the CT system is commissioned to evaluate (1) the breathing signal transfer from the breathing monitoring system to the CT scanner, and (2) the ability to reproduce the test target volume and shape at each breathing stage, as well as the union of individual targets from each breathing stage. We established a baseline for the accuracy of the tests employed and expressed it relative to the reconstructed slice thickness. The proposed tests can be used with any scanner and any acquisition/reconstruction protocol and will result in estimates of the achievable accuracy for an individual system *and* clinical purpose. The periodic QA will merely need to verify that the technical performance of the 4D CT system is maintained and the accuracy level estimated at the time of the commissioning did not degrade.

The proposed commissioning/periodic QA methodology was performed for a newly commissioned CT scanner for different breathing patterns and pitches, and for both phase‐ and normalized amplitude‐based reconstructions, to quantify the accuracy of the reconstructed 4D CT.

## 2 MATERIALS AND METHODS

### 2.1 4D CT equipment and acquisition

All 4D CT image sets were acquired using the Canon Aquilion Exceed LB^13^ scanner (Canon Medical Systems USA, Inc., Tustin, CA, USA) equipped with 80 detector rows with a detector width of 0.5 cm, using an external respiratory signal‐driven low‐pitch helical scanning approach. The CT scanner interfaces with the Anzai (Anzai Medical AZ‐733 V, Anzai Medical, Tokyo, Japan) respiratory gating system equipped with a laser respiration sensor. The Anzai system measures the distance between the laser device and the external patient surface in real time, as a surrogate for the internal movement of the anatomy of interest^14^ and was set to use the inhale peaks to define the beginning of each respiratory cycle. During the reconstruction of the 4D CT acquisition, the respiratory signal from Anzai was used to perform phase‐ and normalized amplitude‐based binning. Ten phase‐binned image sets were reconstructed in 10% increments, ranging from 0% and 90%; 0% and 50% are the labels for the inhale and the exhale image sets, respectively (0% to 50% is the exhalation range, descending from peak to bottom, and 50% to 100% is the inhalation range, ascending from bottom to peak). Likewise, ten amplitude‐binned image sets were reconstructed in 20% increments, ranging from −100% and 80%; −100% and 0% are the labels for the inhale and the exhale image sets, respectively (−100% to 0% is the exhalation range, descending from peak to bottom, and 0% to 100% is the inhalation range, ascending from bottom to peak).

For each acquisition, the Anzai laser detected the motion of a QUASAR™ Respiratory Motion Phantom (pRESP) (Modus Medical Devices, London, Ontario CA) which had an imaging phantom insert that contained a 2.0 cm diameter sphere, which was the subject of the QA. The QA process investigated the scanning system's ability to create accurate 3D digital image sets of the moving sphere. The moving patterns for the sphere were supplied by the respiratory motion software of the QUASAR system, and the sample waveforms are easily reproducible on any motion phantom. Both regular and irregular breathing patterns were used.

After acquisition, the 4D CT scans were imported into MIM (v.7.0.6, MIM Software, Cleveland, OH) for analyses.

### 2.2 4D QA approach

The goal of the 4D CT QA process is to ensure the consistent baseline performance of the system under controlled conditions, representative for common clinical scenarios; a baseline that is established at the time of the commissioning of the system. Of note, a consistently good QA performance is not a guarantee that the patient data will be free of artifacts; instead, it will ensure that no systematic, system‐dependent errors are introduced. This is in line with the general QA approach for any system in radiation oncology. For example, the QA of the on‐board CBCT system guarantees the proper behavior of the imaging system and up‐to‐standard images under specific conditions but does not guarantee artifact‐free images when they are due to causes independent of the imaging system.

The 4D CT commissioning process implemented at our institution comprises of two parts: (1) QA of the accuracy of the motion data transfer between the motion detection system and CT and (2) QA of the accuracy of the geometrical and dynamic characteristics of the imaged target and the derived ITV (volume and shape preservation, and the motion amplitude of the center of mass (COM) of the structures of interest, respectively). The tasks were performed for both normalized amplitude and phase sorting, and for both the regular and the irregular breathing patterns.

The performance of the system was evaluated by comparing the imaged target and the real target and motion characteristics.

For the regular pattern, the scan was performed with several pitch values, including one pitch value matching the breathing rate, and reconstructed with 0.1 cm (only with the pitch setting matching the breathing rate) and 0.3 cm slice thicknesses. For the irregular breathing pattern, the tests were performed for several pitch values, since the CT does not allow for a variable table speed (and thus variable pitch), so it is important to understand how and if the breathing rate during acquisition affects the imaged target if the pitch value deviates from the value assumed at the time the pitch value was set. The normalization of the irregular breathing pattern, used to avoid issues with missing data in amplitude binning, is implemented as follows in the Canon CT software: first, the 100% line is set at the position of the minimum peak (lowest peak) and the 0% line is set at the position of the maximum trough (highest trough); second, all peaks are set to 100% height, all the troughs are set to 0% and the respiratory waveform is rescaled between the peaks and the bottoms accordingly. Our clinical use of amplitude binning is set to employ the normalized breathing data (n‐amplitude).

The proposed QA process is summarized in Table 1. A sample template for tracking and quantifying the QA process is provided (Supporting Information Appendix 1).

**TABLE 1:** Tasks for the 4D CT QA

**QA task**	**Regular and irregular breathing pattern**	
1. Breathing motion characteristics transfer from motion detection system to CT	1a. Breathing pattern comparison. Verify by overlapping the scaled breathing patterns from the breathing detection device and the signal recorded by the CT software	1b. Breathing rate/period verification. Confirm via reading at the CT console
	**Regular breathing pattern**	**Irregular breathing pattern**
2. Target volume accuracy	2a. Compare the nominal target volume with the target structure contoured on each image set	2b. Compare the target ITV (derived theoretically) with ITV derived using the 4D CT
3. Target shape accuracy	3a. Measure displacement of most inferior and most superior aspect of the target on the acquired images and verify that they both match the pre‐set motion amplitude Measure the ITV elongation along the direction of motion and verify that it matches the target ITV dimension derived theoretically	3b. Measure ITV elongation along the direction of motion and verify that it matches the target ITV dimension derived theoretically
4. Target motion amplitude	4a. Measure the COM amplitude of the target motion over the entire 4D image set (0% corresponds to inhale, 50% corresponds to exhale)	4b. Measure the COM amplitude of the target motion over the entire 4D image set (0% corresponds to inhale, but exhale varied from cycle to cycle)

### 2.3 Proposed QA tests

#### 2.3.1 Breathing pattern transfer accuracy from motion detection system to CT (regular and irregular breathing pattern)

##### Breathing pattern comparison

The QUASAR phantom was set in motion to follow a moving (“breathing”) pattern provided by the companion QUASAR computer. The moving pattern of the phantom was recorded by the Anzai laser system, and the shape was compared with the pattern from the QUASAR computer, by overlaying the two waveforms. The patterns were rescaled to the maximum amplitude for easier comparison

##### Breathing period verification

The readout of the breathing period was reported at the Anzai console at the end of each breathing cycle and also at the CT console, as the average of the most recent 30 s of the breathing signal. The breathing rate QA consisted in monitoring the value reported at the CT console and evaluating it relative to the Anzai console value. The QUASAR system time resolution is 0.1 s, and the Anzai system time resolution is 0.01 s. Given the differences between the reporting period of the two quantities of interest, our viewpoint was that a 0.2 s tolerance is reasonable and would not lead to any undesired consequences in the image acquisition process.

#### 2.3.2 Target volume accuracy

The target volume (the sphere) was contoured on multiple reconstructed image sets using phase and n‐amplitude binning, and the corresponding ITV was created for each case. The geometrical characteristics of the target and ITV from the imaging data were compared to the theoretical derived values for the real moving object.

##### Regular breathing pattern

The sphere volume was assessed for all image sets (both phase‐ and n‐amplitude‐binned) at different pitch values, as detailed later in this section.

##### Irregular breathing pattern

Due to the irregular nature of the breathing pattern, there are phases where the volume may be severely distorted and that is expected. Regardless of its quality, the target was contoured on each image set using the same method as the regular breathing pattern and the corresponding ITV was derived. Only the ITV characteristics were analyzed for the irregular breathing patterns.

#### 2.3.3 Target shape preservation

##### Regular breathing pattern

**FIGURE 1:**
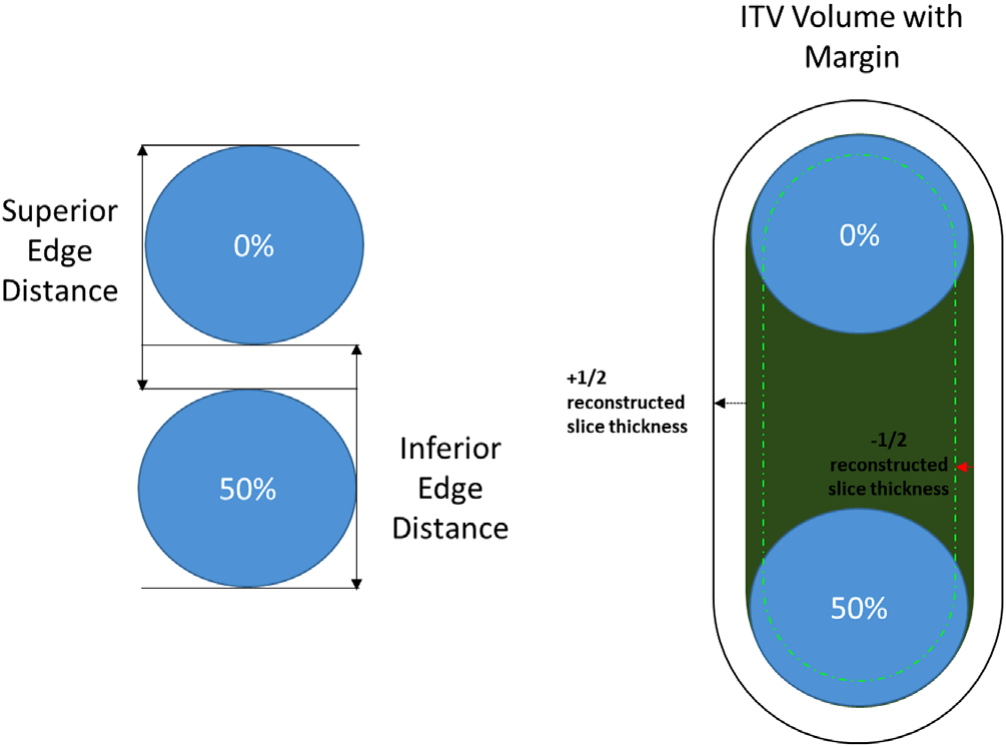
Example of measurements for shape preservation (left) and ITV elongation (right). The shape preservation is the measurement of the inferior and superior border between the inhale (50%) and exhale (0%) breathing cycle. The ITV elongation is the total extent of the target during the full breathing cycle. The margin for the ITV is shown with the upper and lower volume tolerance as a function of the reconstructed slice thickness

To measure the shape preservation of the sphere insert, the distance between the superior and inferior edges of the contour at inhale and exhale were measured (Figure 1). In addition, the elongation of the ITV in the direction the phantom insert's motion (i.e., the distance between the superior and the inferior ITV edges) was measured.

##### Irregular breathing pattern

In the case of irregular breathing, due to the potential missing data or inaccurate information regarding the target volume on each image set, only the accuracy of the ITV elongation in the direction of motion (superior–inferior) was verified and compared to the corresponding theoretical value.

#### 2.3.4 Target motion amplitude

The motion amplitude of the sphere was evaluated as the center‐of‐mass (COM) amplitude of the target motion over the entire 4D image set.

##### Regular breathing pattern

For the sinusoidal respiratory pattern, the extreme locations of the sphere over the 4D cycle occurred at inhale (0%) and exhale (50%), thus the COM of the target at these locations was used to determine the motion amplitude.

##### Irregular breathing pattern

COM amplitude was measured by the same approach used for regular breathing, with the only difference that for the phase reconstruction, the exhale image set did not necessarily correspond to the 50%, therefore, it was identified individually for each 4D image set.

### 2.4 General considerations

#### 2.4.1 Contouring

The sphere volume was contoured and measured in all scenarios with a Hounsfield Units (HU) thresholding tool available in MIM. The threshold value used was 225 and it was determined empirically based on a 3D CT volume HU level that accurately measured the nominal volume of the stationary sphere (4.2 cc). The slice thickness of the 3D scan was 3 mm, the same as the reconstructed slice thickness of the standard clinical scans. The COM was identified and computed using the localization of the contour's centroid option available in MIM.

#### 2.4.2 Pitch selection

The range of allowed pitch values on the Canon Exceed system is [0.026, 0.09], corresponding to breathing rates of [10.0, 2.8] seconds, or [6.0, 21.4] breaths per minute (BPM). For the regular breathing pattern, measurement and analyses were performed for various pitch factors, while keeping the breathing period stable at 4.0 s (15 BPM), as follows: 0.026, 0.043, 0.064, 0.084, 0.09, which are the nominal pitch values for the following breathing rates, respectively: 10, 6, 4, 3, 2.8 s (or, equivalently, 6, 10, 15, 20, 21 BPM). We varied the pitch factor of the table for the regular breathing pattern to investigate the variations in the geometrical characteristics of the target for a range of values between the lower and upper CT scanner technical limits. All analyses were performed for the phase and n‐amplitude binned reconstructions.

For the irregular breathing, four different scans were acquired. The average motion amplitude and the average breathing period during each acquisition are shown in Table 2. The standard deviation of each acquisition is small and for each acquisition a pitch value corresponding to 4 s breathing rate was the closest match, indicating that while the breathing pattern was irregular, the overall phase was consistent between the acquisitions. The pitch was a good match for the average breathing rate only in one of the scenarios, as summarized in Table 2. We varied the pitch factor for the variable breathing pattern because our clinical experience is that the breathing rate during the acquisition may deviate from the breathing rate prior to the scan that constituted the basis for pitch selection.

**TABLE 2:** Acquisition conditions for variable breathing patterns. The average breathing rate during the acquisition and the standard deviation from cycle to cycle is shown along with the average motion amplitude during 4D CT acquisition. Four pitch values were tested, with three of them increasingly deviating from the nominal value corresponding to approximately 4.0 s breathing cycle

**Nominal breaths per minute**	**Acquisition pitch setting**	**Average motion amplitude for the acquisition (cm)**	**Average breathing rate during acquisition (BPM)**	**Standard deviation of the breathing period from cycle to cycle (s)**
15	0.064	1.4	13.3	0.5
10	0.043	1.8	13.9	0.4
7.5	0.033	1.6	14.3	0.5
6	0.026	1.8	13.95	0.6

#### 2.4.3 Considerations regarding the breathing motion amplitude

The regular breathing pattern was a sinusoidal waveform with a nominal amplitude of *A*_n_ = 3 cm and a period *T*_n_ = 4 s. The choice for the motion amplitude was such that it evaluated a less favorable scenario—it has been shown that the distortions due to scanning artifacts are exacerbated when the object size is small in comparison to the extent of motion.^15^ The actual respiratory amplitude of 2.8 cm was less than the amplitude reported by the QUASAR waveform of 3.0 cm; all analyses used 2.8 cm as the actual nominal value.

**FIGURE 2:**
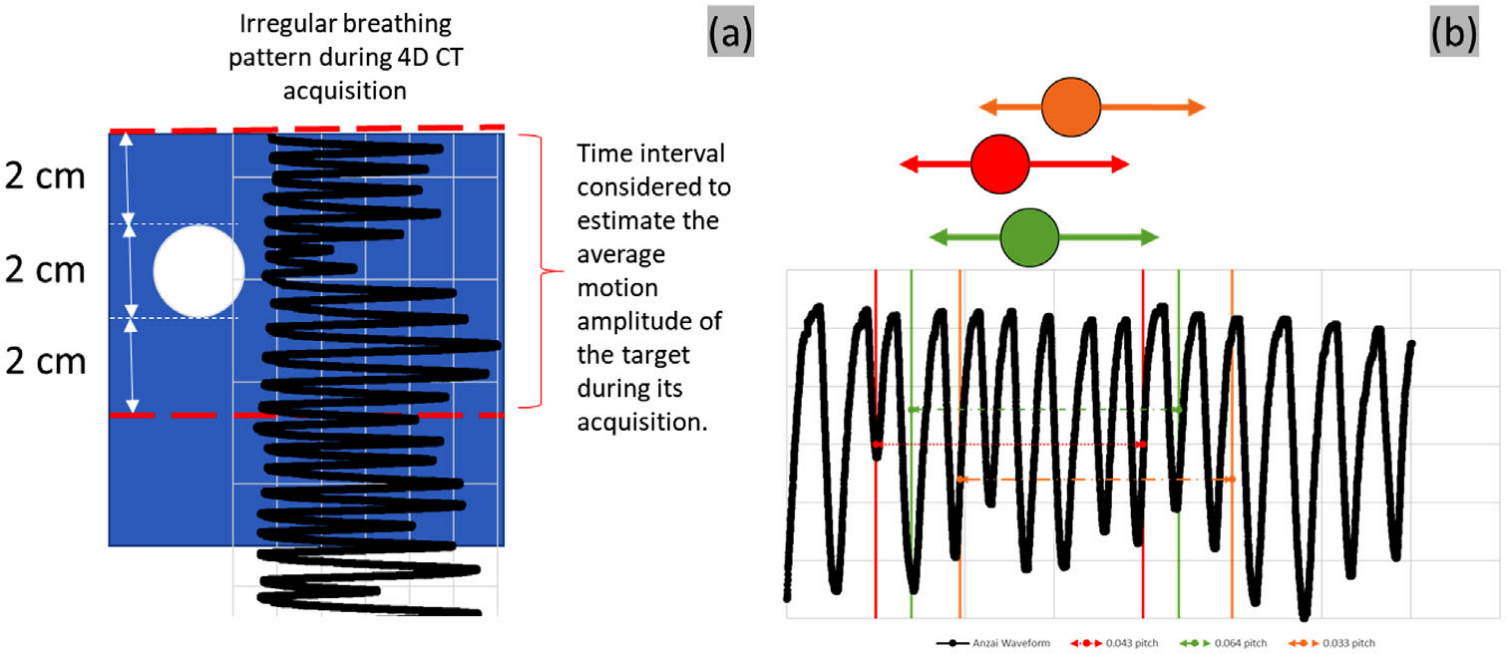
(a) The average motion amplitude of the target was calculated from the breathing pattern recorded ±2 cm above the stationary sphere. (b) The acquired Anzai breathing trace with the different scan acquisition periods along the breathing trace demonstrated for various pitch settings

The irregular pattern had an average phase of 4.5 s over the whole time period of the waveform and an amplitude varying between 1.5 and 2.5 cm. The irregular breathing pattern provided by the QUASAR system far exceeded the time needed for a scan and was replayed continuously during the entire process of acquiring back‐to‐back scans. As such, each 4D scan was acquired under different breathing conditions from the other 4D scan, depending on where the 4D scan on the simulated breathing pattern commenced (Figure 2). In turn, this led to ITV elongations and, consequently, the average motion amplitudes over the target acquisition time that were different from one scan to another (Table 2). The 4D measured results are compared to the scan‐specific average amplitude as determined from the Anzai waveform during the acquisition time that covered the scanning of the target. It is challenging to ensure that the exact period is measured for each acquisition in an irregular breathing pattern. Due to this obstacle we chose to estimate the motion amplitude by taking the average of the phantom position. This average does not represent the average position during the whole extent of the QUASAR irregular pattern, but only the subset that was simulated during the acquisition. Our measured COM is compared to this average that was calculated based on the scan time of each acquisition.

#### 2.4.4 Theoretical ITV values

The theoretical ITV was derived in every case as the volume of a capsule formed by a cylinder whose height depends on the motion amplitude, and two hemispheres, each of radius 1.0 cm (Figure 1). The theoretical extent of the target motion during the irregular breathing was defined as the average elongation over the duration of the acquisition of the moving sphere. The scan limits were set ±2.0 cm around the Cartesian location of the static sphere (Figure 2).

#### 2.4.5 Slice thickness for image reconstruction

For the phase binning, one measurement was also reconstructed at 0.1 cm slice thickness, all other reconstructions were only reconstructed for 0.3 cm slice thickness. For amplitude binning, all reconstructions were performed using normalized respiratory patterns (labeled with (N)).

### 2.5 QA tolerances

Four‐dimensional scans are unique in that the accuracy of the resulting image sets is a combination between the hardware/software performance and the compliance of the imaged object with the requirement of an “ideal” motion pattern (consistently homogeneous in amplitude and periodicity). However, artifacts are unavoidable in 4D, even for regular patterns (due to variable speed of the target), so the goal of the commissioning must be thought in relation to the useful outcome of a 4D scan, which, at the present is, most often, the ITV—the envelope that encompasses the entire motion extent of the target.

The tolerances investigated during QA for each measurement were based on the slice thickness of the *reconstruction*, as the assumed variation in contours and measurements is tied to the thickness of the slices due to volume averaging, with that effect increasing as the slice thickness increases. It is noted that the tolerance for all dimensions of the target is based on the slice thickness which only affects the resolution in the superior–inferior direction. However, the slice thickness has the largest resolution and therefore creates the largest degree of uncertainty. Other clinics may explore tolerances based on a different dimensional characteristic.

All *measured distances* were evaluated by comparison with their respective theoretical values with a tolerance of ±0.5d, where d is reconstructed slice thickness. For all the *volumes*, the tolerances were defined as the volume calculated with defined dimensions with the same tolerances from above.

For example, at a reconstructed slice thickness of 0.3 cm, a measured distance tolerance of ±0.5d is 0.15 cm; for the sphere, the uncertainty spheres have 1.85 cm and 2.15 cm diameters, respectively, and the tolerance volume range is [3.3, 5.2] cc. For the ITV, modeled using a sinusoidal motion of 2.8 cm, it would be a capsule with the nominal volume 9.8 cc the tolerance range [8.2, 11.7] cc (Figure 1).

For certain measurements that did not meet the ±0.5d criteria, we extended our tolerance to ±d and calculated the uncertainty spheres and ITV using the same formulation.

## 3 RESULTS

### 3.1 Breathing pattern transfer accuracy from motion detection system to CT (regular and irregular breathing pattern)

#### Breathing pattern comparison

**FIGURE 3:**
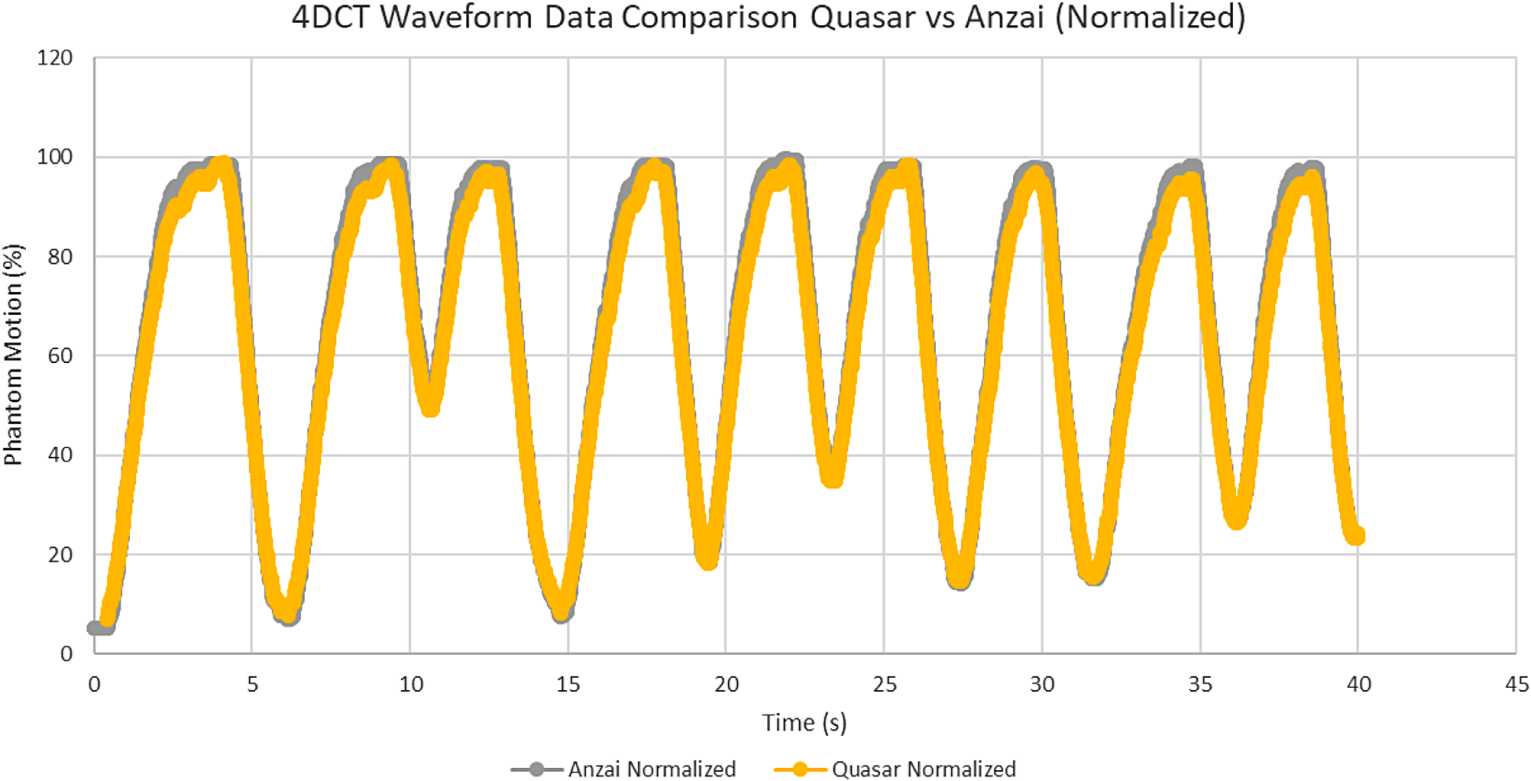
The irregular breathing trace between the QUASAR motion phantom software and the Anzai laser gating system overlaid

The overlays between the motion phantom moving pattern and the displayed Anzai motion pattern were assessed visually and were found to be in agreement. The irregular breathing trace supplied by the QUASAR system to the phantom compared to the trace recorded by the Anzai system is shown in Figure 3.

#### Breathing period verification

The period for the breathing pattern was confirmed at the CT system by verifying that the measured average breath rate acquired by the CT matched the programmed rate of the phantom. All measured deviations were within our tolerance of +/− 0.2 s.

### 3.2 Target volume accuracy

#### 3.2.1 Regular breathing pattern

**FIGURE 4:**
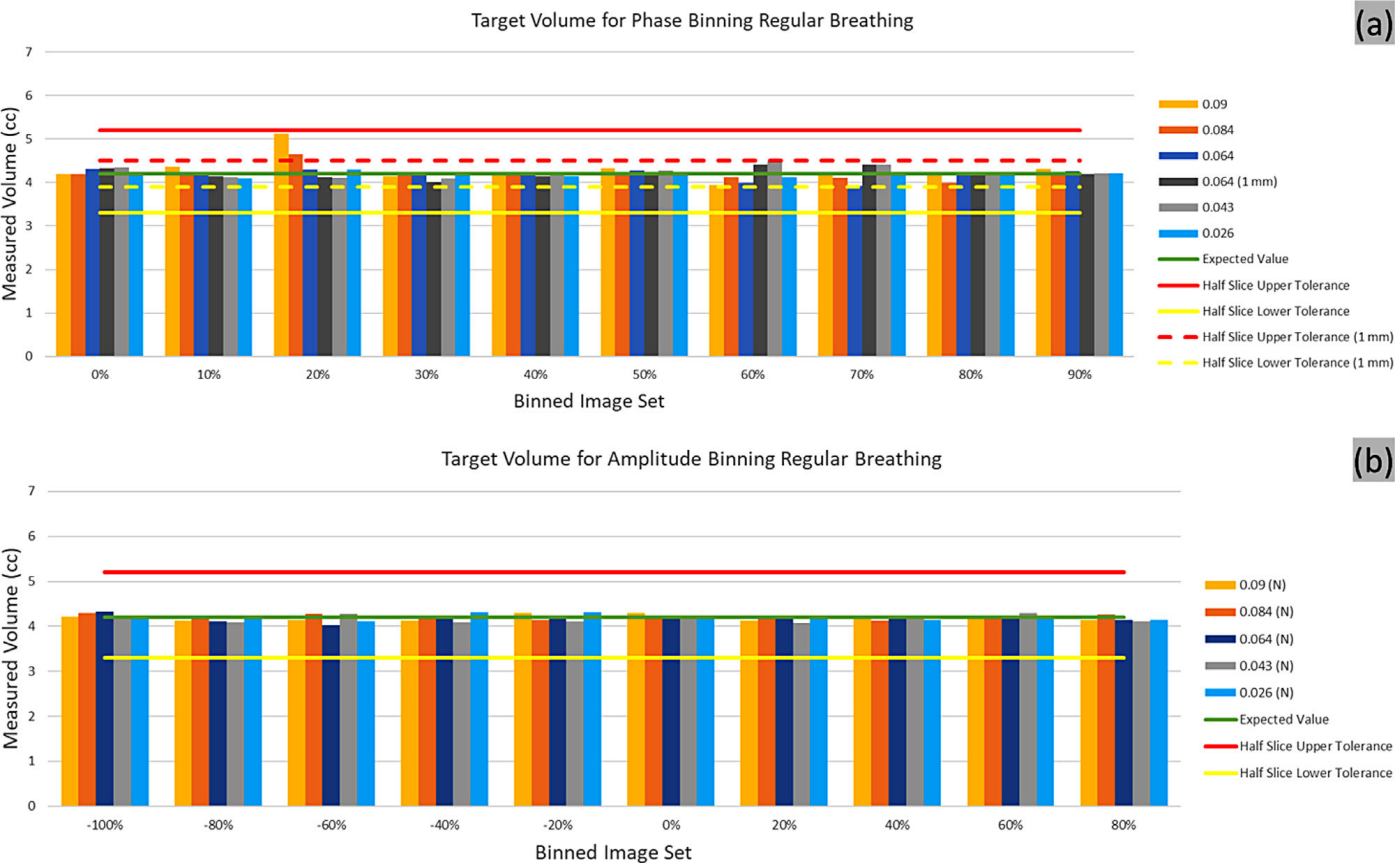
Target volume of the regular breathing for (a) phase and (b) n‐amplitude based binning for various pitch settings. (a) Target volume for phase binning for each pitch setting and a pitch setting reconstructed at 1 mm slice thickness (dark grey). The tolerances based on slice thickness are shown, with an additional set of tolerances (dashed lines) for the 1 mm reconstruction. (b) Target volume for each amplitude binning for each pitch setting. Half slice thickness‐based tolerances are shown

Figure 4 shows the sphere volume for all scenarios under which the 4D CT was acquired, for phase and n‐amplitude binning. The nominal sphere volume (4.2 cc) and the volumes corresponding to the ±half slice thickness tolerance (±0.15/±0.05 cm tolerance) are also shown (solid/dashed line tolerance for 3 mm/1 mm slice thickness).

All volumes except for the one corresponding to the 20% phase reconstruction were well within the tolerance for the corresponding acquisitions, for both the phase and the n‐amplitude binning, as illustrated in Figure 4. The pitch value did not affect the accuracy of the volume, with the lowest accuracy observed for the highest pitch setting (0.09) and phase reconstruction (Table 3). The n‐amplitude binning provided more consistent results (Table 3).

**TABLE 3:** Regular breathing—average/standard deviation of the target volume over all image sets acquired for each pitch value. The nominal target volume is 4.2 cc. The sphere moved with at a 4 s period

	**Phase binning**		**Amplitude binning**	
**Pitch setting**	**Average volume (cc)**	**Standard deviation (cc)**	**Average volume (cc)**	**Standard deviation (cc)**
0.09	4.3	0.3	4.2	0.1
0.084	4.2	0.2	4.2	0.1
0.064	4.2	0.1	4.2	0.1
0.064 (1 mm)	4.2	0.1	–	–
0.043	4.2	0.1	4.2	0.1
0.026	4.2	0.1	4.2	0.1
0.09	4.3	0.3	4.2	0.1

It was noted that for the scenario investigated, the volumes for the 20% reconstructions have larger deviations from the nominal volume at higher pitch values (passing or borderline passing the half slice thickness tolerance). This is not unexpected, considering that at such phase on the inhalation the object moved with higher speed and the higher speed table did not allow for enough scanning time to sufficiently sample the data.

### 3.3 Irregular breathing pattern

**FIGURE 5:**
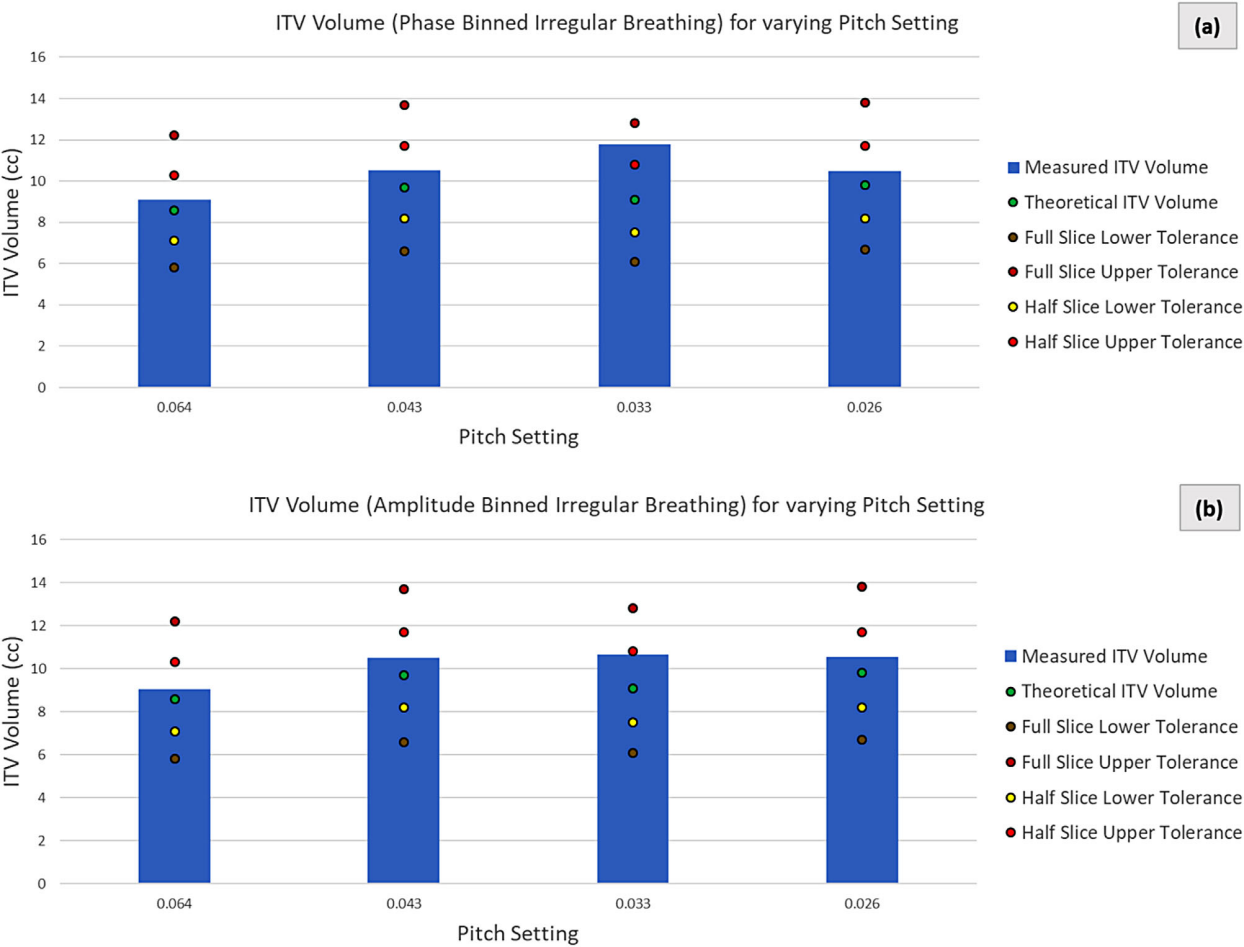
ITVs of the irregular breathing pattern for both phase (a) and n‐amplitude (b) based binned for various pitch settings. The variable theoretical ITV and the resulting tolerances are shown for each pitch setting

Figure 5 shows the measured and the theoretical ITVs, for all irregular breathing acquisitions, along with the respective upper and lower tolerances. There were no notable differences between the phase and n‐amplitude binning. All measured ITVs were within the specified tolerance (except for 0.033 pitch setting with phase binning) and all overestimated the theoretical value computed based on the average motion amplitude over the duration of the 4D CT acquisition. The differences between the measured and the theoretical ITVs averaged (over all pitch conditions) 12.6% and 9.6% for the phase and amplitude binning data, respectively.

The data is inconclusive as far as any potential correlation between the degree of mismatch between the acquisition pitch and the breathing rate.

### 3.4 Target shape preservation

#### 3.4.1 Regular breathing pattern

**FIGURE 6:**
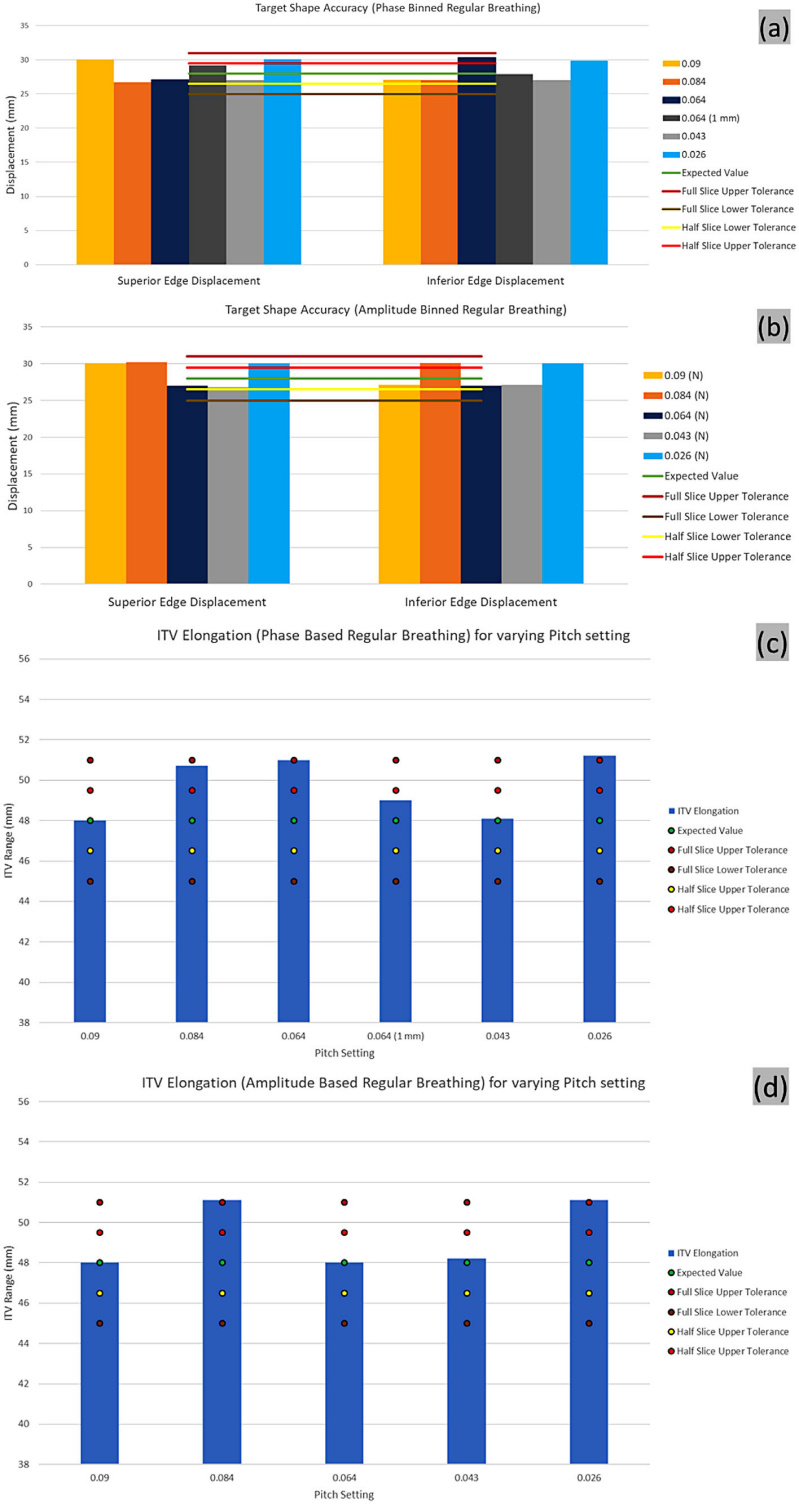
Shape preservation of the regular breathing for (a) phase and (b) n‐amplitude based binning for various pitch settings. Edge distance between breathing phases for 1 mm slice thickness reconstruction is shown (dark grey). Half and full slice (3 mm) thickness‐based tolerances are shown. ITV elongation of the regular breathing for phase (c) and amplitude (d) based binning for various pitch settings. The variable theoretical ITV elongation and the resulting tolerances are shown for each pitch setting

The superior and inferior edge displacements and ITV elongation for the various scans and binning approaches are shown in Figure 6. The 2.8 cm motion amplitude was recovered with negligible deviations in the inferior and superior target edge displacement; however, it appears that deviations were minimized for pitch settings that matched with the breathing rate. ITV elongation agreed well for pitch settings that matched with the breathing rate and for the highest pitch setting. All acquisitions were within the full slice thickness tolerance for the edge displacements. The measured ITV elongation at pitch settings of 0.084 and 0.026 were at or slightly exceed our full slice thickness upper tolerance.

#### 3.4.2 Irregular breathing pattern

**FIGURE 7:**
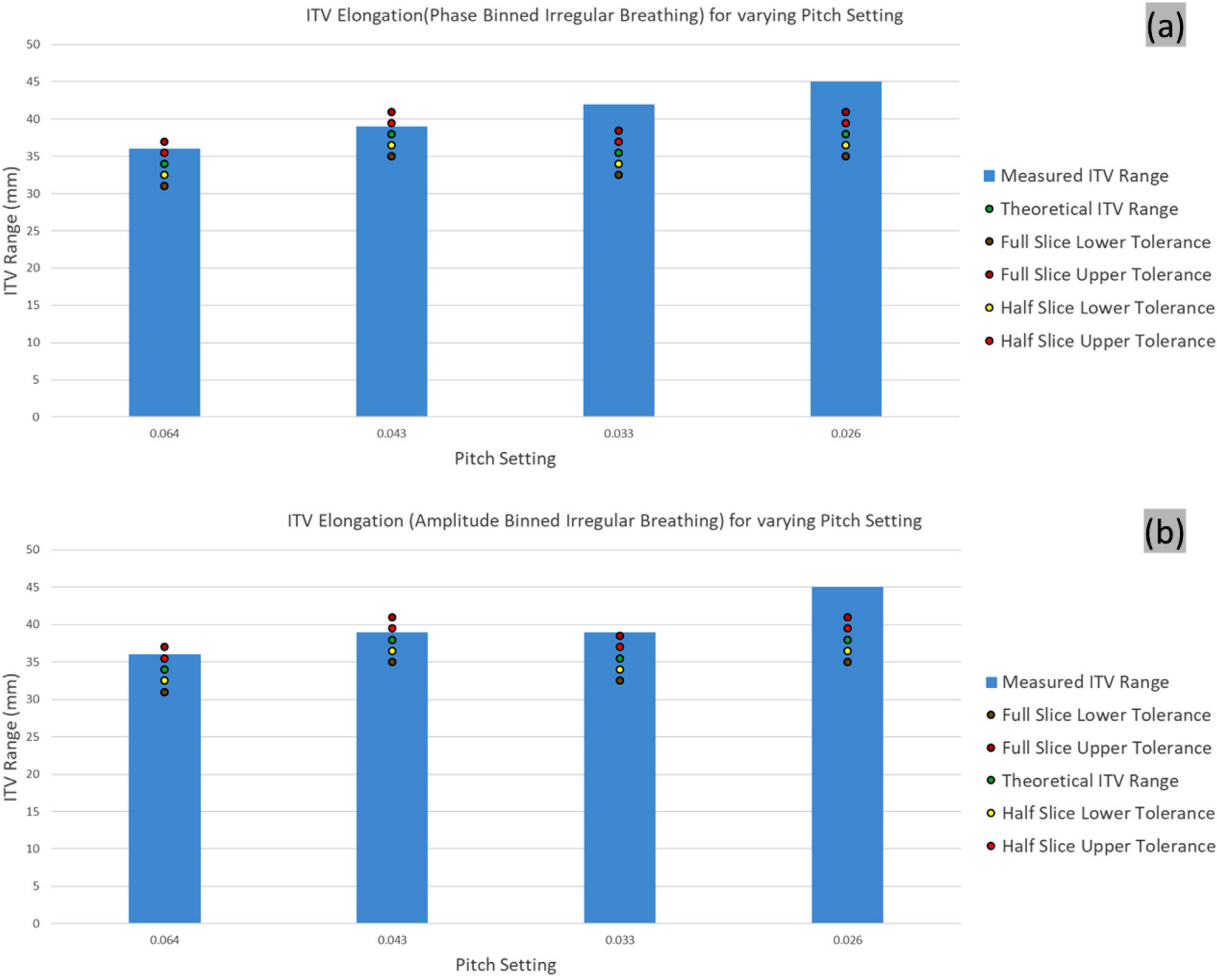
ITV elongation of the irregular breathing for (a) phase and (b) n‐amplitude based binning for various pitch settings. The variable theoretical ITV elongation and the resulting tolerances are shown for each pitch setting

The ITV elongation exceeded the actual average amplitude estimated over the data acquisition, with a larger deviation observed at lower pitch value, with no noticeable differences between the phase and n‐amplitude reconstructions (Figure 7).

### 3.5 Target motion amplitude

#### 3.5.1 Regular breathing pattern

**FIGURE 8:**
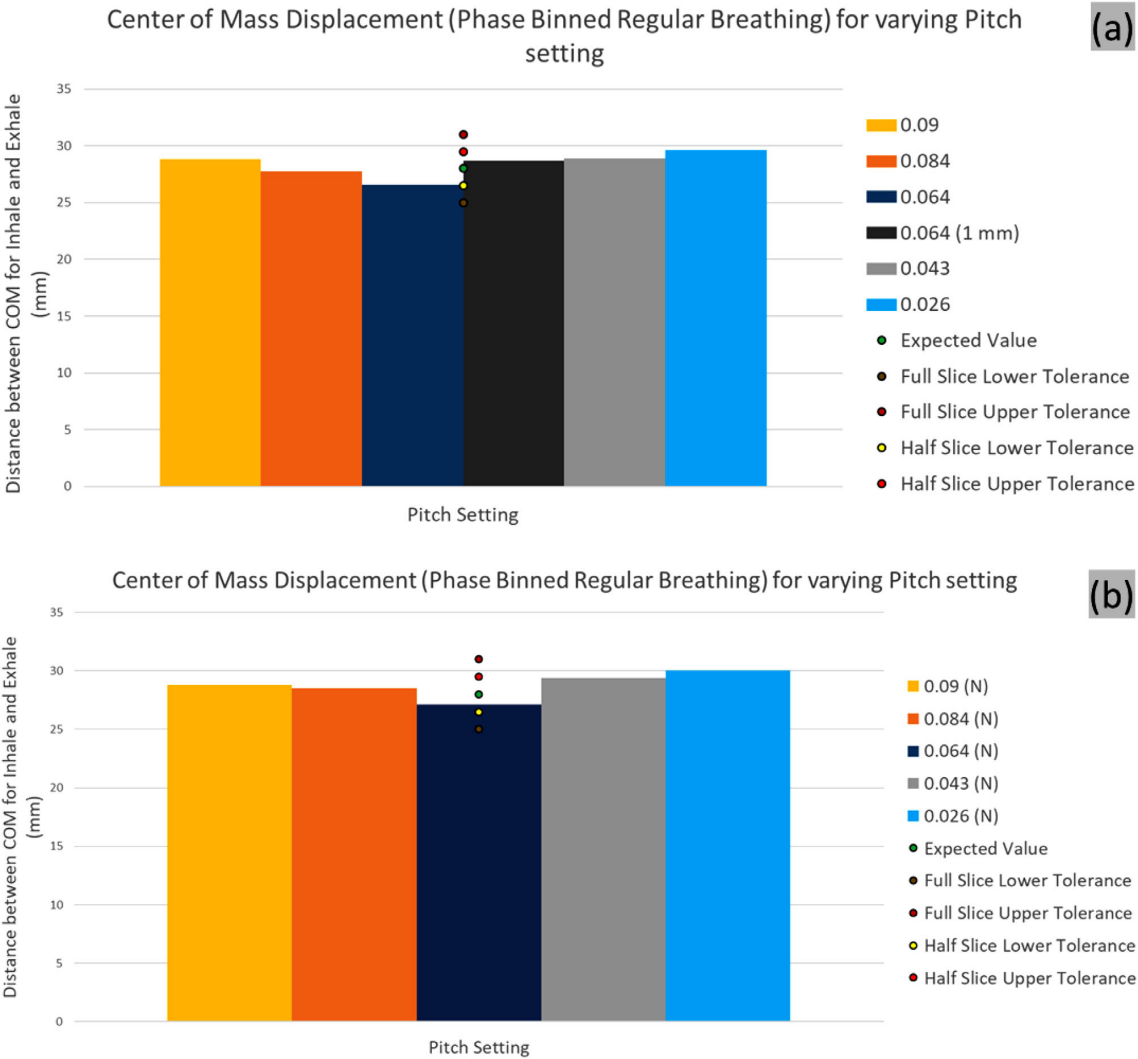
Center of mass (COM) displacement of the regular breathing for (a) phase and (b) n‐amplitude based binning for various pitch settings. COM distance between breathing phases for 1 mm slice thickness reconstruction is shown (dark grey). Half and full slice (3 mm) thickness‐based tolerances are shown

The COM displacement of the target over the breathing cycle was best reproduced by the 0.1 cm slice reconstruction of the scan acquired at the nominal pitch. However, all acquisitions except the lowest pitch setting were able to reproduce the motion amplitude of the COM within half of the reconstructed slice thickness, as shown in Figure 8.

#### 3.5.2 Irregular breathing pattern

**FIGURE 9:**
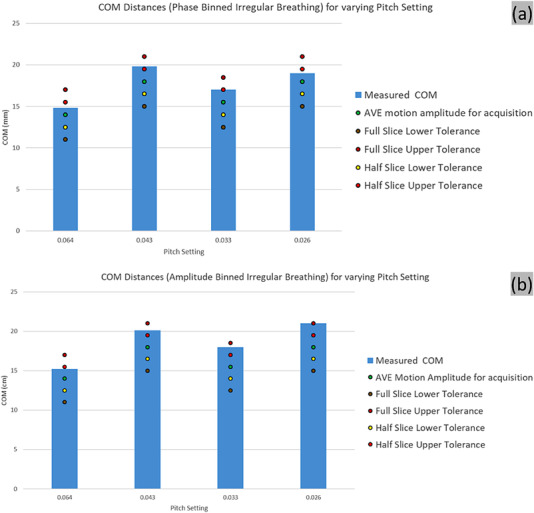
Center of mass (COM) displacement of the irregular breathing for (a) phase and (b) n‐amplitude based binning for various pitch settings. The average motion amplitude for each pitch setting acquisition and the resulting tolerances are shown

The COM displacement was overestimated in all irregular breathing patterns, but the degree of deviation was within the reconstructed slice thickness, in all scenarios. For the examples considered, the phase binning resulted in better COM amplitude match (Figure 9).

## 4 DISCUSSION

Current guidelines exist for the QA of CT simulators dedicated for radiation oncology,^16^ however there are no standardized guidelines or regiments for testing the accuracy of 4D CT equipped CT simulators. Many radiotherapy trials using advanced radiotherapy techniques require rigorous assessment of the accuracy of imaging, delineating, and localization of targets, which can be assessed through film, OSLD, and TLD measurements. However, there are no guidelines that are generalizable to various types of scans and institutions. Recent work by Lambrecht et al. highlight achievable measurements between institutions using the same phantom setup, however they find that there are significant differences in the performance of 4D CT scanning that may be difficult due to differences in institutional protocols and scanner differences.^17^ Additional work has focused on assessing the deviations in 4D CT image quality, particularly in determining the optimal phases for contouring and based on the volume accuracies.^18^, ^19^ We agreed with recommendations to contour GTV while in the exhale phase, though that was not a primary goal of this work. Other studies have noted that sinusoidal motion does not represent normal breathing patterns,^21^ however for our proposed guidelines, it provides value in assessing the accuracy of retrospective gating in a simple and reproducible setup.

Qualitative analysis is sufficient to determine the accuracy of the Anzai system respiratory motion detection system as the breathing signal is only used as a relative measure of the overall amplitude of motion and it is only required that the peak motion is accurately identified to ensure the system captures the motion of the surrogate surface. Note that while the elongation is not needed to reconstruct images, it may be needed for other reasons, for example, to be able to track a tumor during treatment delivery. In such cases, the quantitative verification of the breathing pattern transfer should be included in the commissioning evaluation and period QA.

Another area of interest that has been previously assessed, is the variation in measured amplitude and target size based on variation of pitch.^22^ We did find that lower pitch values capture more accurate ITVs and amplitude, however by choosing the lowest pitch based on the corresponding breathing rate may lead to significant artifacts, particularly between exhale and inhale. While the low pitch may be more clinically acceptable as far as the reduction in deviation from actual tumor motion, it may not be feasible based on the scanner's limitations and lead to shorter scan lengths. We did find that adjusting pitch to be closely aligned with the breathing pattern was acceptable in measuring ITV and range for irregular patterns. This framework can be used to assess the accuracy of the 4D CT scanner reconstruction of ITV for variable breathing patterns but will be unable to determine how a scanner will operate under clinical conditions.

Even while using the thresholding tool there will still be variation in the measurement of the target shape's volume. To assess the accuracy of the 4D CT reconstruction we based our volume tolerance on the slice thickness, which has been investigated for contouring accuracy. Prionas et al., in a phantom study of CT scan parameters effect on volume contouring, found that volumes were always under‐measured, with increasing error with decreasing sphere diameter, and increasing error with increasing slice thickness.^23^ However, they found diminished returns in using progressively smaller slice thickness though the coefficient of variation decreased with decreasing slice thickness. Because of these findings we recommended using a sphere of at least 1.0 cm diameter in measurements and a slice thickness that corresponds to the clinical setting and basing the tolerance on half of the slice thickness when assessing performance with the pitch setting matched to the phantom motion, though this has not been assessed rigorously. In assessing less ideal acquisitions where the pitch setting does not correspond with the phantom motion a higher tolerance up to the full slice thickness is sufficient. It appears that for acquisitions with 1 mm slice thickness the accuracy may be even greater, as we did not find any issues meeting the regular breathing tolerances.

Regarding the accuracy of amplitude versus phase binning various authors previously found that amplitude is more accurate.^8^, ^24^, ^25^ We found that to be similarly true based on the volume accuracy for binned volumes (Figure 4b). It appears that the phase volumes were larger than the actual value, particularly during phases where the degree of motion is greatest (20%‐30%). However, as shown previously, amplitude binning can be prone to missing data, which we see in the higher pitch factors at amplitudes around −60% to 40%. While normalization can prevent issues due to missing data that can cause issues with reconstruction, or it may lead to misplaced volumes. In our acquisitions, both phase and n‐amplitude binning fared well for the sinusoidal breathing pattern, and the variability is well accounted for with a tolerance of ±0.5 the slice thickness for individual phase targets and for the ITV.

It is accepted that an irregular breathing pattern acquisition is needed for rigorous QA,^26^ and we have employed an irregular pattern that varied in the range of 1‐2 s in the breathing period and had a variation of the amplitude on the order of 1‐2 cm at points. These variations, while they may not encompass all clinical scenarios, will assess the scanner ability to reconstruct more difficult 4D CT patient studies.

Our tests showed that under normal breathing conditions, the target volume was accurately captured in the 4D CT acquisition, even when there was a mismatch between the employed pitch values and the breathing rate. The ITV for the irregular breathing pattern exceeded the nominal value in all scenarios and was similar for both the phase and the n‐amplitude binning. The mismatch between the nominal pitch and the actual breathing rate during acquisition did not seem to affect substantially the size of the ITV. In all cases the measured ITV exceeded the theoretical ITV. The COM amplitude was captured accurately in the case of the regular breathing and overestimated in the irregular breathing scenarios.

The regular breathing test returned accurate data, providing confidence that the hardware, the software, and the input–output between the systems perform adequately. The irregular breathing data, as part of the initial system commissioning, aimed at evaluating the potential systematic deviations that would need to be accounted for during the planning and delivery of the radiation treatment. While no test can broadly and properly predict and investigate the entire variability that can be encounter with patient data, our commissioning tests suggest that the system may be expected to capture in excess the target motion and geometry, but the deviation is expected to be within the slice thickness. Therefore, an additional margin to account for the 4D acquisition inaccuracies does not seem necessary with the system tested, when using pitch settings close to the measured breathing rate. It was also noted that the pitch value did not seem to affect in any detrimental way any of the geometrical characteristics that have been quantified.

Our data also demonstrated that the geometrical accuracy in the case of the breathing pattern outperforms that irregular breathing one. This underlines the importance of the motion mitigation strategies that would make the breathing pattern more consistent in both phase and amplitude.

## 5 CONCLUSIONS

In this study we have presented a robust commissioning framework that assesses the performance of the 4D CT scanning for both regular and irregular breathing patterns. We used a regular breathing pattern to evaluate the relevant 4D‐specific hardware, the software and the input–output between the systems perform adequately. The irregular breathing data was used to evaluate the geometrical properties of the ITV, which is the most consequential 4D CT derivate used in treatment planning and delivery.

We proposed tolerances based on the slice thickness of the acquisition and the scanner exceeded the tolerance in all cases of the regular breathing pattern, even when varying the table pitch. The implementation of the proposed approach can be tailored to the specific clinical needs of any given clinic. The regular breathing provided confidence that the hardware, the software, and the input–output between the systems performs adequately. The irregular breathing data suggest that the system may be expected to capture in excess the target motion and geometry, but the deviation is expected to be within the slice thickness. Therefore, an additional margin to account for the 4D acquisition inaccuracies does not seem necessary with the system tested. It was also noted that the pitch value did not seem to affect in any detrimental way any of the geometrical characteristics that have been quantified. Our data also demonstrates that the geometrical accuracy of the regular breathing pattern outperformed the accuracy of the irregular breathing pattern.

## AUTHOR CONTRIBUTIONS

Mitchell Polizzi contributed to study design, data collection, analysis, and writing of the manuscript. Siyong Kim contributed to study design, supervision of the project, and writing of the manuscript. Mihaela Rosu‐Bubulac contributed to study design, analysis, supervision of the project, and writing the manuscript. All authors discussed the results and contributed to the final manuscript.

## Supporting information

Supporting Information
