# Supplementary material for: A comprehensive quality assurance procedure for 4D CT commissioning and periodic QA
Source: J Appl Clin Med Phys. 2022 Sep 4;23(11):e13764. doi: 10.1002/acm2.13764 (PMC9680562; doi:10.1002/acm2.13764)
Supplement: Supplementary file 1 — Supporting Information [file ACM2-23-e13764-s001.pdf]

1) CT Data

Manufacturer	______	
Model	______	
Device Serial Number	______	

2) Scan Parameters

Slice Thickness (d)	______	mm
Rotation Time	______	mm
Total Beam Collimation Width	______	mm
Pitch Factor	______	
HU Threshold	______	HU

3) Regular Motion and Volume Parameters

Motion Phase (P_{reg})	______	sec
Phantom Amplitude (A_{reg})	______	mm
Phantom Insert Radius (r)	______	mm

4) Irregular Motion Parameters

Max Amplitude (during acquisition)	______	mm
Average Phase (P_{irreg})	______	sec
Average Amplitude (during acquisition) (A_{irreg})	______	cm

5) 4DCT Reconstruction Parameters

Phase Reconstruction	to	%
Bin Size	______	%
Amplitude Reconstruction	to	%
Bin Size	______	%

Breathing Motion Characteristics Transfer Tolerances

6. Breathing Rate Verification

b.	Lower Tolerance	______	sec
c.	Upper Tolerance	______	sec

Regular Breathing Pattern Tolerances

7. Regular Breathing Target Volume Accuracy

	Theoretical Volume (V_{reg})	$((4/3)\pi(r)^3)$	______	cc
c.	Volume Lower Tolerance	$((4/3)\pi(r-d/2)^3)$	______	mm
d.	Volume Upper Tolerance	$((4/3)\pi(r+d/2)^3)$	______	mm

8. Regular Breathing Target Shape Accuracy

b.	Lower Tolerance	$(A_{reg}-d/2)$	______	mm
c.	Upper Tolerance	$(A_{reg}+d/2)$	______	mm
d.	Theoretical ITV Elongation (L_{reg})	$(A_{reg}+2r)$	______	mm
e.	ITV Elongation Lower Tolerance	$(A_{reg}+2r)-d/2$	______	mm
f.	ITV Elongation Upper Tolerance	$(A_{reg}+2r)+d/2$	______	mm

9. Regular Breathing Motion Amplitude

b.	Lower Tolerance	$(A_{reg})-d/2$	______	mm
c.	Upper Tolerance	$(A_{reg})+d/2$	______	mm

Irregular Breathing Pattern Tolerances

10. Irregular Breathing Target Volume Accuracy

c. Theoretical ITV Volume (ITV_{irreg}) ______ cc

$$(A_{irreg}\pi(r)^2)+(\frac{4}{3}\pi(r)^3)$$

d. ITV Volume Lower Tolerance ______ cc

$$(\pi r^2(A_{irreg}-\frac{d}{2}))+(\frac{4}{3}\pi(r-\frac{d.}{2})^3)$$

e. ITV Volume Upper Tolerance ______ cc

$$(\pi r^2(A_{irreg}+\frac{d}{2}))+(\frac{4}{3}\pi(r+\frac{d}{2})^3)$$

11. Irregular Breathing Target Shape Accuracy

c.	ITV Elongation (L_{irreg})	$(A_{irreg}+2r)$	______	mm
d.	Lower Tolerance	$(A_{irreg}+2r)-d/2$	______	mm
e.	Upper Tolerance	$(A_{irreg}+2r)+d/2$	______	mm

12. Irregular Breathing Motion Amplitude

b.	Lower Tolerance	$(A_{irreg}-d/2)$	______	mm
c.	Upper Tolerance	$(A_{irreg}+d/2)$	______	mm

Regular Breathing Pattern QA

Spiral Pitch Factor

**Binning Mode:**					**2d.**
**Breathing Motion Characteristics Transfer**					
**QA Test Condition**	**Measured Value**	**Expected Value**	**Lower Tolerance**	**Upper Tolerance**	**Result**
Breathing Pattern Comparison	N/A	N/A	N/A	N/A	
Breathing Rate [seconds]		P_{reg}	6b.	6c.	

Target Volume Accuracy

QA Item	Measured Value	Expected Value	Lower Tolerance	Upper Tolerance	Result
Regular Respiration, 0%		V_{reg}	7c.	7d.	
Regular Respiration, 10%					
Regular Respiration, 20%					
Regular Respiration, 30%					
Regular Respiration, 40%					
Regular Respiration, 50%					
Regular Respiration, 60%					
Regular Respiration, 70%					
Regular Respiration, 80%					
Regular Respiration, 90%					

Target Shape Accuracy

QA Item	Measured Value	Expected Value	Lower Tolerance	Upper Tolerance	Result
Superior Edge Distance between inhale and exhale		A_{reg}	8b.	8c.	
Inferior Edge Distance between inhale and exhale		A_{reg}	8b.	8c.	
ITV Elongation		L_{reg}	8e.	8f.	

Target Breathing Motion Amplitude

QA Item	Measured Value	Expected Value	Lower Tolerance	Upper Tolerance	Result
COM distance between inhale and exhale (0% and 50%)		A_{reg}	9b.	9c.	

Irregular Breathing Pattern QA

Binning Mode:	Spiral Pitch Factor<br>2d.

Breathing Motion Characteristics Transfer

QA Test Condition	Measured Value	Expected Value	Lower Tolerance	Upper Tolerance	Result
Breathing Pattern Comparison	N/A	N/A	N/A	N/A	
Breathing Rate		P_{irreg}	6b.	6c.	

Target Volume Accuracy

QA Item	Measured Value	Expected Value	Lower Tolerance	Upper Tolerance	Result
ITV Volume		ITV_{irreg}	10d.	10e.	

Target Shape Accuracy

QA Item	Measured Value	Expected Value	Lower Tolerance	Upper Tolerance	Result
ITV Elongation		L_{irreg}	11d.	11e.	

Target Breathing Motion Amplitude

QA Item	Measured Value	Expected Value	Lower Tolerance	Upper Tolerance	Result
COM distance between inhale and exhale		A_{irreg}	12b.	12c.	
